# Modelling covalent linkages in *CCP*4

**DOI:** 10.1107/S2059798321001753

**Published:** 2021-05-19

**Authors:** Robert A. Nicholls, Robbie P. Joosten, Fei Long, Marcin Wojdyr, Andrey Lebedev, Eugene Krissinel, Lucrezia Catapano, Marcus Fischer, Paul Emsley, Garib N. Murshudov

**Affiliations:** a Structural Studies, MRC Laboratory of Molecular Biology, Francis Crick Avenue, Cambridge CB2 0QH, United Kingdom; b Netherlands Cancer Institute, Plesmanlaan 121, 1066 CX Amsterdam, The Netherlands; c Oncode Institute, The Netherlands; d Global Phasing Limited, Sheraton House, Castle Park, Cambridge CB3 0AX, United Kingdom; e CCP4, STFC Rutherford Appleton Laboratory, Chilton, Didcot OX11 0QX, United Kingdom; f Randall Centre for Cell and Molecular Biophysics, Faculty of Life Sciences and Medicine, King’s College London, London SE1 9RT, United Kingdom; g Chemical Biology and Therapeutics and Structural Biology, St Jude Children’s Research Hospital, 262 Danny Thomas Place, Memphis, TN 38105-3678, USA

**Keywords:** covalent linkages, *AceDRG*, *CCP*4, monomer library, link records, link dictionary, mmCIF, restraints, CCP4 Monomer Library, link-restraint dictionary

## Abstract

In this contribution, the current protocols for modelling covalent linkages within the *CCP*4 suite are considered. The mechanism used for modelling covalent linkages is reviewed: the use of dictionaries for describing changes to stereochemistry as a result of the covalent linkage and the application of link-annotation records to structural models to ensure the correct treatment of individual instances of covalent linkages. Previously, linkage descriptions were lacking in quality compared with those of contemporary component dictionaries. Consequently, *AceDRG* has been adapted for the generation of link dictionaries of the same quality as for individual components. The approach adopted by *AceDRG* for the generation of link dictionaries is outlined, which includes associated modifications to the linked components. A number of tools to facilitate the practical modelling of covalent linkages available within the *CCP*4 suite are described, including a new restraint-dictionary accumulator, the *Make Covalent Link* tool and *AceDRG* interface in *Coot*, the 3D graphical editor *JLigand* and the mechanisms for dealing with covalent linkages in the *CCP*4*i*2 and *CCP*4 *Cloud* environments. These integrated solutions streamline and ease the covalent-linkage modelling workflow, seamlessly transferring relevant information between programs. Current recommended practice is elucidated by means of instructive practical examples. By summarizing the different approaches to modelling linkages that are available within the *CCP*4 suite, limitations and potential pitfalls that may be encountered are highlighted in order to raise awareness, with the intention of improving the quality of future modelled covalent linkages in macromolecular complexes.

## Abstract 2

The mechanism for modelling covalent linkages in *CCP*4 is reviewed and the method of link-dictionary generation used by *AceDRG* is described. An overview of the various protocols available for the modelling and application of covalent linkages within the *CCP*4 suite is presented, providing instructive guidelines with a focus on practical application.

## 1. Introduction   

Modelling covalent interactions between compounds requires special consideration during macromolecular model building and refinement. In addition to requiring knowledge of the particular atoms that are covalently bound, it is necessary to have a complete chemical description of the system (including bond orders *etc.*) as well as a corresponding restraint dictionary that describes the local geometry, along with any modifications to either of the linked compounds.

Challenges typically encountered when modelling covalent linkages include detecting the presence of a covalent linkage, identifying the correct chemistry and obtaining appropriate restraints for use in refinement (Kleywegt, 2007 ▸; Zheng *et al.*, 2014 ▸; Koval’ *et al.*, 2019 ▸). General mechanisms for generating and applying restraints between covalently bound components have existed for decades. The two main approaches that have been used involve full local atom-typing (Tronrud *et al.*, 1987 ▸; Engh & Huber, 1991 ▸; Brünger, 1992 ▸) and the linking of larger individual monomers (Vagin *et al.*, 2004 ▸). In both cases the large number of potential chemical configurations has proven to be prohibitive, with detailed link dictionaries only being available for commonly occurring chemistries (for example polymeric linkages).

The CCP4 (Winn *et al.*, 2011 ▸) Monomer Library (CCP4-ML), also referred to as the REFMAC5 Dictionary (Vagin *et al.*, 2004 ▸; Murshudov *et al.*, 2011 ▸), contains a number of component and link dictionaries. For an overview of the current status of the CCP4-ML, see Nicholls *et al.* (2021 ▸). In addition to distributing a number of pre-computed descriptions in the CCP4-ML, there is also a need to facilitate the *ad hoc* generation of custom link dictionaries, as well as the ability to easily and/or automatically ensure that covalent linkages are correctly applied to a given model.

The procedure involved in the generation and application of bespoke covalent linkages has been awkwardly confusing and error-prone, often involving expert knowledge and/or requiring manual file editing. The lack of tools to facilitate and automate this process has resulted in manual consideration being required in a large number of cases. Failure to provide a comprehensive restraint dictionary representing a covalent linkage often results in just a single interatomic distance restraint being applied between linked components; this is insufficient to ensure good resultant model geometry. This has undoubtedly negatively affected the quality of links in many deposited models and caused the inconsistent treatment of analogous chemistries across different Protein Data Bank (PDB) entries (Berman *et al.*, 2007 ▸). It is known that covalent binding affects the stereochemistry of neighbouring atoms, yet modifications to local chemistry have typically not been sufficiently accounted for when describing linkages. This has resulted in inappropriate geometric restraints for the surrounding environment and thus sub­optimal refinement of many macromolecular complexes or, at least, varying quality and consistency of geometric restraints in the immediate vicinity of modelled covalent linkages.

Existing tools for the generation of link dictionaries include *grade* (Smart *et al.*, 2011 ▸), which generates *TNT*-style link dictionaries (Tronrud, 1997 ▸) suitable for use with *BUSTER* (Bricogne *et al.*, 2017 ▸), and *WriteDict*, part of *AFITT* (OpenEye Scientific Software; Wlodek *et al.*, 2006 ▸), which is integrated into *Phenix* (Janowski *et al.*, 2016 ▸). The *Phenix* suite (Liebschner *et al.*, 2019 ▸) also includes *REEL* (Moriarty *et al.*, 2017 ▸) to facilitate the manual editing of restraints output by *eLBOW* (Moriarty *et al.*, 2009 ▸). Previously, the recommended approach to link generation in *CCP*4 involved the use of *LibCheck* (Vagin *et al.*, 2004 ▸) using *JLigand* (Lebedev *et al.*, 2012 ▸), often via *Coot* (Emsley *et al.*, 2010 ▸). However, the ability to routinely generate suitably comprehensive restraint dictionaries for covalently linked components, of a quality akin to that of contemporary ligand-dictionary generation technology, has been unavailable to date. In response to this deficiency, *AceDRG* (Long *et al.*, 2017 ▸) has recently been extended to allow the generation of dictionaries for describing covalent linkages.

In Section 2 we review the conventional approaches to modelling covalent linkages in *CCP*4: the use of link records for annotating particular instances of a linkage within an atomic model and of restraint dictionaries for describing a type of linkage. Section 3 discusses the approach to link-dictionary generation implemented in *AceDRG*. Section 4 summarizes the tools currently available for modelling covalent linkages in the *CCP*4 suite. Both *Coot* (Section 4.5) and *JLigand* (Section 4.6) have been modified to allow *AceDRG* to be used for link-dictionary generation; these are the preferred routes when using *CCP*4 *Cloud* (Krissinel *et al.*, 2018 ▸; Section 4.8). Recent developments in *Gemmi* (Wojdyr, 2017 ▸), exposed in the *CCP*4*i*2 (Potterton *et al.*, 2018 ▸) *Make Covalent Link* interface (Section 4.7), aim towards providing a more robust user experience. Practical examples are provided in Section 5.

Throughout this article we specifically focus on the implementation and tools available within the *CCP*4 suite; analogous tools are available from other suites. Some of the tools and resources discussed, notably the CCP4-ML, *AceDRG*, *REFMAC*5 and *Coot*, are also distributed as part of the *CCP-EM* suite (Burnley *et al.*, 2017 ▸); many features discussed here in the context of macromolecular crystallography can also be directly transferred to electron cryo-microscopy.

We shall refer here to a ‘model’ as meaning a structural atomic model, unless otherwise stated.

## 2. Conventional approaches to modelling covalent linkages in *CCP*4   

In this section, we shall reflect on the usage of link records and restraint dictionaries for describing covalent linkages, according to implementations within the *CCP*4 suite. In order to model a covalent linkage, it is necessary to provide a connectivity annotation (*i.e.* a link record) that specifies for a particular atom pair within the model to be treated as covalently bound. Also, a separate link-description dictionary is required which specifies the chemical connectivity and geometric restraints associated with a particular linkage (including references to any required modifications to the bonded compounds). Whilst not technically a strict requirement, such dictionaries are highly recommended in order to avoid poor resultant model geometry; thus, they should be considered as a requirement in modern application.

Link records are only needed for nonstandard bonds. For example, they are not required for peptide or phosphodiester linkages between adjacent residues, which are defined in the CCP4-ML. It should be noted that peptide bonds involving a noncanonical amino acid such as selenomethionine (MSE) or phosphothreonine (TPO) are also recognized by *REFMAC*5 without the need for link records. This holds true for any peptide bond between two monomers categorized as ‘peptide’ (any amino-acid residue with standard backbone-atom naming) in the CCP4-ML; the equivalent applies to nucleotides with the group name ‘DNA’ or ‘RNA’. There are 509 amino-acid and 270 nucleotide components in the CCP4-ML that are linked automatically. Indeed, any linkages that have descriptions in the CCP4-ML are automatically created and applied during refinement by *REFMAC*5 if the potentially linked atoms have the same chain identifier^1^ and are sufficiently proximal or are consecutive in sequence numbering. Note that this is the same mechanism as used for the automatic application of polymeric linkages (for example between amino acids in a polypeptide chain, nucleotides in nucleic acids and saccharides in carbohydrate chains).

For more detailed discussion and annotative examples of covalent linkages and modifications, see Lebedev *et al.* (2012 ▸), and for formal definitions, see Vagin *et al.* (2004 ▸).

### 2.1. Link-annotation records in PDBx/mmCIF files   

PDB Exchange (PDBx; Deshpande *et al.*, 2005 ▸), which is derived from the Macromolecular Crystallographic Information Framework (mmCIF; Fitzgerald *et al.*, 2006 ▸), is the preferred contemporary format for model storage. In fact, submission of PDBx/mmCIF files is now a mandatory requirement upon deposition in the wwPDB (Adams *et al.*, 2019 ▸). These files allow the recording of any supplementary connectivity information (in the struct_conn data category; Bourne *et al.*, 1997 ▸), including link records. Such records specify the presence of covalent bonds between compounds, for example due to post-translational modifications.

A *CCP*4 variant of the PDBx/mmCIF format allows the optional specification of a particular link identifier (via the CCP4_link_id data item) that uniquely references the full link description, which may be found in the CCP4-ML or in a custom dictionary. Any information regarding link identifiers is not currently used by the OneDep system at the point of deposition (Young *et al.*, 2017 ▸). To clarify, link identifiers are only used internally by software such as *REFMAC*5 during the model-building and refinement process. Since the link identifiers are discarded upon deposition, information regarding the exact chemistry and modelling assumptions made when refining the model is also lost at the point of deposition.

### 2.2. Link-annotation records (LINK) in PDB files   

In PDB files, covalent linkages have traditionally been handled using LINK records (Callaway *et al.*, 1996 ▸), noting that disulfide bridges, which are very common, are considered a special case and are instead treated using SSBOND records. For technical details, see Vagin *et al.* (2004 ▸) and Lebedev *et al.* (2012 ▸).

LINK records merely indicate that there is a bond between particular atoms. They are not meant to specify refinement targets, and simply state that there is a bonding interaction. The PDB format prescribes that LINK records include a ‘link distance’, which should be set to the current interatomic distance between the linked atoms (taking potential symmetry operations into account). This ‘link distance’ is typically ignored during refinement (see below for practical exceptions), although exactly how this information is interpreted and utilized is implementation-specific; this is a common cause of confusion.

In *REFMAC*5, if a LINK record is specified in the absence of a corresponding dictionary entry to describe that covalent linkage, then only a single covalent-bond restraint is applied between the two atoms. If the atom types are present in the CCP4-ML, with a corresponding restraint representing their bonding, then that restraint is used. Determining the appropriate stereochemistry, and thus the appropriate restraint, can be difficult, especially in the absence of explicitly modelled H atoms; this may potentially result in inappropriate restraints. If a matching restraint is not available in the CCP4-ML (for example for many metal-involving atom pairs) then a restraint is generated with a target value equal to the ‘link distance’ reported in the LINK record. If the link distance is absent then *REFMAC*5 calculates a default target value based on the covalent radii of the atoms.

Either way, if a restraint dictionary is not available then only a single interatomic distance restraint is used to represent the covalent linkage. This means that other geometric properties (for example inter-component angles) that represent the local structural configuration are not restrained. However, such restraints are recommended in order to ensure that, for example, the relative orientation of the linked components is reasonable. In addition, modifications to the internal restraints for each of the involved components are not applied; the effect of this can be dramatic, especially when the covalent linkage results in chemical changes within the components (for example changes in bond orders or the addition or removal of atoms). Consequently, compared with the use of a detailed dictionary, this typically results in an atomic model of suboptimal quality (Nicholls *et al.*, 2021 ▸).

### 2.3. Extended link-annotation records with identifier extension (LINKR) in PDB files   

**Figure 1:**
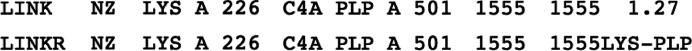
Example LINK and LINKR records, corresponding to the covalent linkage of the NZ atom in lysine (LYS) and the C4A atom in pyridoxal phosphate (PLP). In this case, LYS-A226 is linked to PLP-A501. The two fields marked ‘1555’ correspond to symmetry operators (in this case the linked atoms are located within the same asymmetric unit). LINK and LINKR records differ only in the final field: LINK records have a link distance (for example ‘1.27’), whereas LINKR records have a link identifier (for example ‘LYS-PLP’). For further details about the format of LINK records, see Callaway *et al.* (1996 ▸).

One problem with standard formal PDB LINK records is that they do not allow the specification of the exact nature of a given linkage. For example, LINK records do not encode information regarding bond order, nor whether any chemical modification of either compound is required as a result of the covalent bonding. Hence, there is potential ambiguity regarding the chemistry, and thus which dictionary should be used to define linkage geometry. In such cases, the decision regarding which dictionary to use (if indeed such a dictionary even exists) is left up to the downstream refinement software. Consequently, *REFMAC*5 accepts a variant of the PDB format that has an extended LINK record, which allows the specification of a link identifier in place of the link distance (see Fig. 1 ▸). This link identifier explicitly references a particular link description, which may be located in the CCP4-ML or in a custom dictionary. For clarification of the format variant, such extended records are marked as LINKR instead of LINK^2^; we shall here refer to the extended version as LINKR, in order to make this distinction clear. *REFMAC*5 preferentially uses records with a link identifier where possible; using this approach allows a complete description of the correct linkage chemistry and any modifications to the linked components, along with the associated restraints. This is equivalent to specifying a link identifier in *CCP*4 variant PDBx/mmCIF files.^3^

### 2.4. Restraint dictionaries   

Restraint dictionaries are used to describe the connectivity and geometry of molecular components (Vagin *et al.*, 2004 ▸). These dictionaries are based on the mmCIF format, which is a macromolecular specialization of the more general CIF format (Hall *et al.*, 1991 ▸) that can be used to store many types of crystallographic data (Brown & McMahon, 2002 ▸). In the present context, restraint dictionaries are required to describe each constituent component of the model; these individual component types (for example amino-acid residues, nucleotides, ligands, waters *etc.*) are identified by a unique component identifier, which in current practical usage is treated as synonymous with ‘residue name’, ‘monomer id’ and ’three-letter code’.^4^ These ‘component dictionaries’ specify the chemical nature of each of the constituent atoms (element, charge), the way in which the atoms are bonded (bond order, aromaticity) and additional chemical/geometric properties (orbital hybridization, chirality), as well as any restraints produced by the dictionary-generation software, for example representing interatomic bonds, angles, torsion angles and planes, along with associated estimated standard uncertainties.

In addition to those for the individual components, dictionaries describing all modelled covalent linkages between components are also required. Whilst analogous in format to component dictionaries, these ‘link dictionaries’ are distinct in terms of content. They comprise two facets.

(i) The description of the covalent linkage itself: references to the components and atoms to be linked and the qualitative nature of the bond, along with associated distance, angle, torsion and planar restraints.
(ii) Descriptions of the modifications that need to be applied to each of the dictionaries of the linked components in association with the particular covalent linkage, including any changes to the atomic composition (for example removing atoms), connectivity, chemical properties and geometric restraints of the individual components.

Both link records and modification records are assigned their own identifiers, which must be unique and self-consistent in order to avoid ambiguity; link descriptions cross-reference particular component modifications by their identifiers. Note that there may be multiple modifications that could be applied to a given component, and there may be multiple link types that use the same modification. Indeed, there is a separate link description for each chemical linkage type. There may theoretically be multiple link descriptions corresponding to the bonding of a given atom pair between two particular residues that correspond to different chemistries; for example, differing bond orders of the covalent linkage (and implied changes to protonation) and/or differing modifications to be applied to the chemical composition/properties of either of the linked components. In the case of such ambiguities, *REFMAC*5 selects the first matching link entry. Consequently, it is important that the connectivity annotation record within the model references the correct identifier for the corresponding link dictionary; it is worth being mindful of such considerations when using link dictionaries.

Note that it may be necessary to reuse component and link dictionaries both within and between models; a given model may exhibit multiple instances of the same covalent linkage, and different models may exhibit the same local chemistry. For example, there are 4469 instances of the α-1,3-glycosidic linkage, which is the covalent bond between the O3 and C1 atoms of pyranose components, amongst 1740 PDB entries (up to 36 link instances per model). Another example is the covalent linkage between LYS[NZ] and PLP[C4A] (see Fig. 5), of which there are 1598 instances modelled amongst 792 PDB entries (up to 12 link instances per model).

In order to facilitate reusability, component/link dictionaries are usually located in separate files from the model. Pre-computed dictionaries corresponding to many of the most commonly occurring components and link types, including the α-1,3-glycosidic linkage, are distributed as part of the CCP4-ML. The CCP4-ML has recently seen substantial expansion, including the addition of link dictionaries for commonly occurring covalent linkages, including LYS[NZ]–PLP[C4A] (Nicholls *et al.*, 2021 ▸). Custom dictionaries must be generated for any other components and link types encountered, in which case it is important to ensure that such bespoke dictionaries maintain uniqueness and self-consistency of component, link and modification identifiers.

### 2.5. Restraint-dictionary accumulation   

Each individual restraint dictionary (whether for component, link or modification) may be physically located in separate files or accumulated into an aggregate dictionary. Due to the format compatibility of PDBx/mmCIF model and restraint dictionaries, any dictionary information used during refinement may be additionally encapsulated when using the PDBx/mmCIF model format (for the purposes of completeness and tracking the provenance of utilized prior knowledge).

However, since *REFMAC*5 only allows a single custom dictionary to be provided as input, it is necessary for dictionaries to be accumulated prior to model refinement. Where multiple dictionaries are used, it is necessary to ensure that they do not conflict in order to avoid potential ambiguity and error. In response to this need, a new tool to facilitate dictionary accumulation is now available in *CCP*4, which performs validation in order to ensure the compatibility of dictionary entries and includes the ability to automatically reassign modification identifiers where necessary.^5^ These tools utilize the *Gemmi* library for structural biology (Wojdyr, 2017 ▸).

## 3. Covalent link-description generation using *AceDRG*   

In this section, we shall discuss the approach to link-dictionary generation implemented in *AceDRG* (Long *et al.*, 2017 ▸). *AceDRG* was primarily designed for the creation of ligand-description (component) dictionaries from a simple chemical description, as well as the generation of initial coordinates corresponding to a low-energy conformer. *AceDRG* has recently been extended to allow the generation of link dictionaries, using the same fundamental procedural principles as for component-dictionary generation.

The introduction of a covalent bond between two monomers affects the internal chemistry and geometry of each of the two components. Consequently, instead of attempting to treat the two monomers and the link independently, *AceDRG* considers the composite component complex as a whole and generates a dictionary for this complex as if it were a single monomer. The end result is that the linkage is modelled as if it were a natural part of one larger hypothetical molecule, and thus the resultant link dictionary contains geometric restraints derived from detailed information regarding the local chemical and structural environment (up to the third order).

Whilst the specific details vary, in essence the procedure is analogous to that used by *JLigand* for the creation of link dictionaries using *LibCheck* (as described by Lebedev *et al.*, 2012 ▸). Specifically, link-dictionary generation with *AceDRG* involves three stages, which are detailed in the three subsequent subsections.

(i) Construction of an initial composite component: a hypothetical molecule comprising the two components to be connected by a covalent linkage (Section 3.1).
(ii) Derivation of a detailed stereochemical description of the composite component, including information about bond lengths, angles, torsions, chiral centres, rings and planar groups (Section 3.2).
(iii) Qualitative and quantitative comparison of the geometric descriptions of the individual and composite components. Differences between these descriptions are included in the output link dictionary (Section 3.3).

Examples of the practical application of *AceDRG* link dictionaries are provided in Section 5.

### 3.1. Construction of an initial composite component   

*AceDRG* reads and processes instructions regarding the covalent bond between two monomers. Such instructions include the specification of the atoms that are to be covalently linked, the bond order of the linkage and any chemical modifications to any of the atoms in either component (for example changes in atomic composition, charge or bond orders; see Fig. 5). Given such a chemical specification, *AceDRG* firstly sanitizes the valences of the linked atoms to report any possible gross errors such as valency violations. This sanitization involves adding/deleting bonded H atoms to/from the linked atoms in order to achieve the required valency. If the valency must be reduced but there are no bonded H atoms, *AceDRG* will adjust the formal charges of the atoms as necessary. Where multiple valences are possible, for example for sulfur and boron, the option that would involve minimal modification is selected. Once all necessary modifications have been applied and validated, the bonding pattern of the composite compound is constructed and the whole composite compound is sanitized.

### 3.2. Geometric description generation for the composite component   

Given the bonding graph of the composite component, *AceDRG* generates a stereochemical dictionary using the procedure described by Long *et al.* (2017 ▸). This results in a composite component dictionary containing geometric restraints. A low-energy conformer is also generated, representing one potential conformation of the hypothetical composite molecule.

### 3.3. Identification of differences between individual and composite component dictionaries   

The dictionaries corresponding to the individual and composite components are compared in order to identify any differences. Any intra-component differences are described as modifications to the individual components. The two original components are assigned their own modification records, with unique identifiers. Any inter-component information found in the composite dictionary is assigned to a link record, with a given link identifier. This link identifier should be referenced wherever an instance of the particular linkage type occurs within a model (as discussed in Section 2). Note that the link record internally references the modification records, so they are automatically used whenever the link identifier is referenced. Modifications are applied in the order in which they are presented. The resultant link dictionary comprises both the link record and the two component-modification records. If one or other of the input compound descriptions is not in the CCP4-ML then the corresponding component dictionaries are also added to the output file. Example link dictionaries are provided as supporting information.

## 4. Current tools for modelling covalent linkages in *CCP*4   

In this section, we discuss different approaches to modelling covalent linkages, focusing on practical application. We firstly discuss the merits and drawbacks associated with replacing individual residues with larger composite components, rather than modelling them as individual covalently linked compounds (Section 4.1). We outline how the link dictionaries available in the CCP4-ML are automatically used where possible (Section 4.2) and highlight the importance of using component identifiers that correctly reflect the implied chemistry (Section 4.3). We then give an overview of modern tools within the *CCP*4 suite for the generation and application of link records and dictionaries, specifically *AceDRG*, *JLigand* and *Coot* (Sections 4.4–4.6 [Sec sec4.5] [Sec sec4.6]) and the *Make Covalent Link* task in *CCP*4*i*2 (Section 4.7), as well as a discussion of the flow of information pertaining to covalent linkages in *CCP*4 *Cloud* (Section 4.8). It should be noted that each of the interfaces for dictionary generation discussed in Sections 4.5–4.8 [Sec sec4.6] [Sec sec4.7] [Sec sec4.8] use *AceDRG*; each of these workflows should involve the creation of identical link dictionaries.

**Figure 2:**
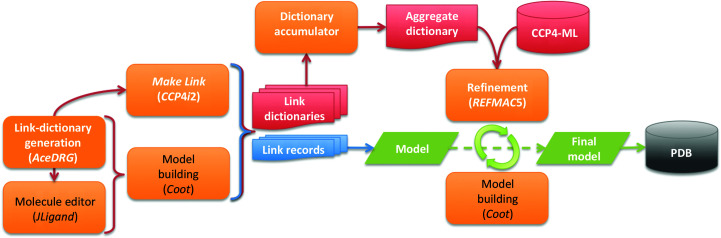
Dataflow involved in modelling covalent linkages using the *CCP*4 suite, as coordinated by the graphical project-management environments *CCP*4*i*2 and *CCP*4 *Cloud*. Processes and programs are depicted as orange rectangles, data as document symbols (arrays indicate the potential presence of multiple instances), models as parallelograms and databanks as cylinders. Arrows indicate directional flow, coloured according to the matching data type: red for link dictionaries, blue for link records and green for models. Text labels are coloured black for graphical interactive processes and white for (semi-)automated processes and data. Additional representations are provided as supporting information: see Supplementary Fig. S1 for a simplified linkage information dataflow and Supplementary Fig. S2 for a GUI-centric process flow diagram.

Fig. 2 ▸ depicts a general abstraction of the dataflow involved in modelling covalent linkages with *CCP*4. *AceDRG* is the recommended tool for link-dictionary generation. *AceDRG* may be executed from within *Coot* or *JLigand* (via *Coot*); these are the recommended routes when using *CCP*4 *Cloud*. *AceDRG* can also be executed from a command-line interface, as well as via the *Make Covalent Link* task in *CCP*4*i*2. Both *Coot* and the *Make Covalent Link* task can add link records to a model; the latter of these can scan a given model for matching instances of a linkage and apply link records accordingly (maintaining the appropriate identifiers). In cases where there are multiple custom restraint dictionaries (for components, links *etc*.), they must be accumulated into a single aggregate dictionary, ensuring internal consistency and uniqueness of nomenclature and identifiers. This aggregate dictionary, along with any required dictionaries from the CCP4-ML, is used by *Coot* and *REFMAC*5 during the iterative model-building and refinement process. The final model deposited in the wwPDB contains link records, but without link identifiers.

### 4.1. Replacing individual residues with larger composite components   

Treating linked components as a single larger entity, and generating a new component dictionary for that composite component, is a technically valid option. There are examples of this within the PDB, one such component being LLP, which represents the linked LYS–PLP complex (as modelled, for example, in PDB entry 1ajs; Rhee *et al.*, 1997 ▸). For the specifics of this example, see Lebedev *et al.* (2012 ▸). Previously, the main benefit of such a composite component approach was to ensure that the restraints for the internal geometry would be of the same quality as for individual components (in contrast to the use of a simple LINK record). However, due to having a different component identifier, any other linkages (for example polymeric linkages) involving the composite component would have to be re-specified, resulting in unnecessary duplication and potential for error. Another problem with this approach is that explicit references to the individual components (in this case LYS and PLP) are lost; such information could be useful in subsequent downstream analysis.

Fortunately, there is no longer a need to replace residues with larger composite components in order to model covalent linkages, as tools are now available that allow the routine creation of quality link descriptions. The modern architecture promoted within this article, which involves linking smaller components together, is more general and flexible than requiring the availability of explicit dictionaries for larger composite components.

However, there are a number of cases where a complex has traditionally been treated as a modified component rather than modelling the covalent linkage between two components (for example phosphotyrosine, which has the component identifier PTR). In such cases, it is important to follow the typical convention in order to avoid extra work upon deposition of the final model in the wwPDB. Replacing a residue by its modified counterpart can be performed efficiently with the ‘replace residue’ tool in *Coot*. For lists of commonly occurring modified amino acids and otherwise special components, see Table 1 in Lebedev *et al.* (2012 ▸) and Table 2 in van Beusekom *et al.* (2021 ▸).

The composite component complex approach may also be required in difficult cases, such as when there are multiple linkages between the same two components or when a link dictionary involves more than two components (a use case not currently supported by modern dictionary generators).

Note also that the use of the linkage mechanism should be restricted to describing the result of chemical reactions in which two components become covalently bound: this has a clear biological interpretation. Other geometric restraints that involve multiple residues, for example hydrogen-bond restraints from *ProSMART* (Nicholls *et al.*, 2013 ▸) or *HODER* (van Beusekom, Touw *et al.*, 2018 ▸), should be defined as external restraints for *REFMAC*5 and *Coot*. Whilst it is acceptable to use modification records to describe minor changes to internal component chemistry (for example deletion of an atom, change of formal charge *etc.*), they should not be used to describe excessive changes to internal component chemistry. Indeed, it is important to ensure that both components to be linked are modelled using appropriate monomer descriptions before attempting to model the covalent linkage between them.

### 4.2. Automatic application of linkages from the CCP4-ML   

For standard linkages present in the CCP4-ML, software such as *REFMAC*5 and *Coot* automatically detect and apply linkages to a model based on the proximity of atoms. When multiple link dictionaries are available that match a given atom pair (in the CCP4-ML and/or a custom restraint dictionary), *REFMAC*5/*Coot* must decide which dictionary to use. In such cases, the dictionary with the most detailed matching specialization will be selected; exact matches are preferred over wildcard entries, and conformational analysis may be performed in cases where there are multiple exact matches. However, note that any such potential ambiguities are avoided if the model contains connectivity annotation records that specify exactly which link dictionary should be used for each particular instance (as discussed in Section 2).

An example that stresses the importance of using correct link identifiers can be found with glycosidic linkages. The large number of related carbohydrates allows a generalization of linkages between pyranoses. Each type of linkage has α and β anomeric types that differ only in the chirality around the C1 atom. In order to refine with the correct restraints and avoid distortion of the linkage geometry, the correct link identifier must be specified based on the expected stereochemistry. Some degree of automation was achieved previously with the *PDB-REDO* (Touw *et al.*, 2016 ▸) program *stripper* (and its replacement *prepper*) that set the correct link identifier in the coordinate files based on an extendable dictionary of 48 common pyranose–pyranose linkages before being passed to *REFMAC*5 (van Beusekom, Lütteke *et al.*, 2018 ▸).

### 4.3. Ensuring the correctness of compound identities   

As part of the process of the correct application of covalent linkages and the efficient use of existing descriptions in the CCP4-ML, an important step is ensuring the use of the correct residue nomenclature. Even when two monomers seem to be identical, it is always important to use the one with the correct identifier, *i.e.* the one that corresponds to a dictionary with the correct chemical composition, stereochemical connectivity and atomic nomenclature, especially when constructing linkages. A straightforward example is adenosine monophos­phate, which exists both as a standalone ligand (identifier AMP) and as part of an RNA polymer (identifier A). As long as the correct residue name is used, *REFMAC*5 and *Coot* will use the correct linkage restraints without the need to add specific link-record annotation.

**Figure 3:**
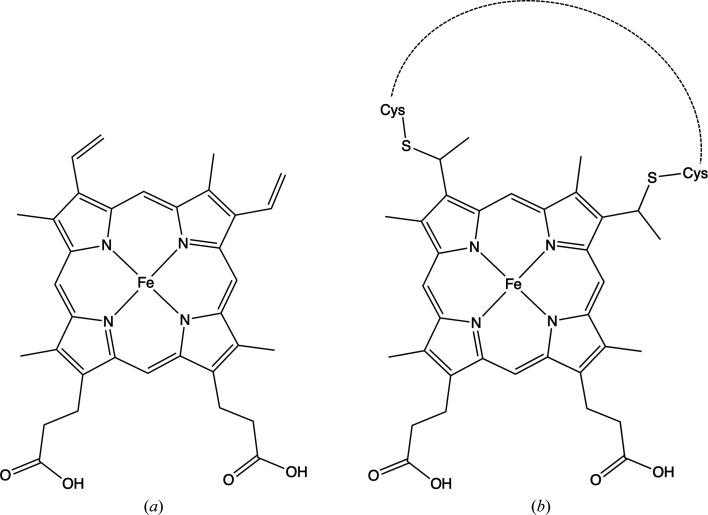
Modelling haem B and haem C using monomer descriptions from the Chemical Component Dictionary (CCD; Westbrook *et al.*, 2015 ▸). (*a*) Haem B (CCD identifier HEM). (*b*) Haem C (CCD identifier HEC) covalently bound to protein via cysteine thiols. Note that the wwPDB recommends against using the CCD component HEM for modelling haem C, which is found covalently linked to other components via thioether bridges. Also, the Fe atom should have charge +2 (unless bound to another molecule); standard representations are presented. Images were created using *ChemDraw Professional* 17.1.

In some cases special care is required when selecting the appropriate component identifier for a particular compound. Haem groups are an example of this (see Fig. 3 ▸). Haem B (HEM) does not make covalent bonds to cysteine (CYS) side chains, whereas haem C (HEC) does (Takano *et al.*, 1977 ▸). For an example, see PDB entry 4ub6 (Suga *et al.*, 2015 ▸), in which both haem B and haem C are modelled (HEM E103 and HEC V201). Rather than generating link descriptions between HEM and CYS, the wwPDB recommendation is to rename the compound HEC and use the appropriate link descriptions already available in the CCP4-ML (identifiers ‘HEC-CYS1’ and ‘HEC-CYS2’; the associated modifications change the bond orders appropriately). The *PDB-REDO* program *prepper* performs this automatically when HEM is modelled as being bound to CYS or when a cysteine thiol is within 2.5 Å of the appropriate C atom in a haem. A survey of the PDB using *prepper* revealed 754 cases in which HEM residues, instead of HEC, were used to model haem C. Similarly, there are 112 PDB entries in which HEC is inappropriately modelled as a standalone (noncovalently bound) ligand.

### 4.4. *Make Link* tool in *Coot*   

The simple *Make Link* tool in *Coot* (located in the Modelling menu) produces and adds a standard LINK record to a model (see Section 2.2). It does not produce a link dictionary. Consequently, there is no control over the exact nature of the implied chemistry. *Coot* now checks whether an appropriate link dictionary is available and will generate a warning if there is not.

The use of this tool may suffice for a common post-translational modification, for which there is an unambiguous corresponding entry present in the CCP4-ML. However, when applying just a simple LINK record there is the danger of uncertainty about treatment during downstream refinement (as discussed in Section 2.2). Consequently, the recommended contemporary approaches for linkage generation in *Coot* are the *AceDRG* link interface and *JLigand*.

### 4.5. *AceDRG* link interface in *Coot*   

An interface to the link-dictionary generation functionality has recently been added to *Coot* (version 0.8.9.1). This is found in the *CCP*4 module (which is activated in the Calculate menu, under Modules). The *CCP*4 module contains a menu item *Make Link via AceDRG* which opens a dialogue that asks for the following.

(i) The bond order corresponding to the covalent linkage (default: single).
(ii) Which non-H atoms should be deleted (if any).
(iii) Which bond orders change as a result of this new linkage (if any).

The user then clicks on the two atoms to be linked. *Coot* executes *AceDRG* to produce the required link dictionary, which is then imported into *Coot* so that it is available for subsequent real-space refinement. On successful reading of a link dictionary, *Coot* provides visual feedback by representing the new linkage as a dotted line between the linked atoms.

### 4.6. Creating link dictionaries using *JLigand*   

*JLigand* was originally designed as a graphical interface for *LibCheck*, allowing users to visually create and edit chemical graphs for ligands and produce component and link dictionaries, as well as generate regularized coordinate models. *JLigand* is now able to use *AceDRG* for component- and link-dictionary generation; *AceDRG* is recommended over *LibCheck*. However, *LibCheck* can still be used as a contingency in cases that *AceDRG* cannot presently handle (*i.e.* metals).

*JLigand* is closely integrated with *Coot*: following the selection of two atoms in *Coot*, *JLigand* is launched displaying the two components to be linked. *JLigand* can then be used to specify the details of the covalent linkage (for example bond order, component modifications *etc.*). The link dictionary is then generated and communicated back to *Coot*, at which point *Coot* generates and applies the corresponding link record to the model. *JLigand* provides a more interactive graphical alternative to the *AceDRG* interface of *Coot*.

Although *JLigand* uses a mechanism similar to *AceDRG* when generating link dictionaries (as described in Section 3), the specific implementation is different and thus the results may differ in some cases. In addition, *JLigand* imposes no restrictions on the degree to which the components to be linked may be internally edited; care should be taken, as it provides no warning in the case of excessive modifications to the components and no guidance on whether the link description being generated already exists in the CCP4-ML.

### 4.7. Dealing with covalent linkages in *CCP*4*i*2   

The *CCP*4*i*2 GUI for macromolecular crystallography (MX) project management (Potterton *et al.*, 2018 ▸) allows the results from one job to be easily passed as input to another, using data abstraction to focus on data objects as opposed to raw files. This ability to transfer necessary objects from one task to another facilitates and expedites the iterative model-building and refinement procedure. Close integration with *Coot* allows data objects created within *Coot* to be transferred back to the *CCP*4*i*2 project for subsequent downstream use, including custom restraint dictionaries comprising component and link descriptions. Indeed, *AceDRG* dictionaries created via *Coot* can be reused elsewhere in a *CCP*4*i*2 project, and vice versa.

Recently, the *Make Covalent Link* task has been implemented in *CCP*4*i*2 to facilitate the creation of *AceDRG* link dictionaries and their application (*CCP*4 version 7.1). Descriptions for the two components to be linked are required: these may be automatically imported from the CCP4-ML using the relevant three-letter code or from a custom component dictionary. Where required, such dictionaries can be created separately using *AceDRG* via the *Make Ligand* task. The interface automatically inspects the component dictionaries in order to determine the lists of atoms within the two components. After selecting the atoms to be linked, and specifying the linkage bond order, the user may also select to optionally delete atoms, change bond orders and change formal charges within each of the components.

This task may be used in isolation in order to make an abstract link description, or it can be used in conjunction with a particular model. In the latter case, the model is searched for all potential instances of the specified linkage type, according to proximity criteria. The user may then select whether to automatically apply link records for all identified potential instances of the linkage, or to add just one link record for a specific instance.

The *Make Covalent Link* task utilizes the *Gemmi* library (Wojdyr, 2017 ▸) to search the CCP4-ML for available components, to inspect atoms and bonds in component dictionaries, to search a model for matching instances of a given linkage and to apply link records to the model.

### 4.8. Dealing with covalent linkages within the *CCP*4 *Cloud* environment   

*CCP4 Cloud* provides a data-driven GUI that assembles all associated metadata, including references to data files, into an object called a ‘structure revision’ (Krissinel *et al.*, 2018 ▸). A series of revisions accumulate data during the structure-determination process so that by the time the project is at the stage of model refinement, the current revision incorporates a variety of information, including reflection data, the expected macromolecular sequence, the atomic model and a dictionary containing any bespoke restraints for ligands and covalent linkages. This approach allows effortless bookkeeping and thus, hopefully, a seamless user experience.

In a particular revision, the dictionary of restraints includes accumulated descriptions of ligands and linkages created in any *Model Building with Coot* and *Fit Ligand with Coot* tasks that were previously run in that particular branch of the project tree (restraint dictionaries may be imported, generated using the *Make Ligand* task or created in *Coot*). Thus, any component and link dictionaries generated during, for example, one *Coot* job are naturally accessible and used in any subsequent *REFMAC*5 and *Coot* jobs.

When dealing with linkages for a particular atom pair, the *Coot* task in *CCP*4 *Cloud* performs different actions depending on the presence of a dictionary for that linkage type. If a link description is not present then *Coot* inserts a standard LINK record into the output coordinate file (see Section 2.2). However, if an appropriate link dictionary is available in the structure revision then a LINKR record is used instead, which contains an explicit reference to the correct link identifier in the dictionary (see Section 2.3); this automated mechanism provides a fluent workflow.

## 5. Examples of modelling covalent linkages using *AceDRG* dictionaries   

The link dictionaries generated for the examples presented in this section are provided as supporting information.

### 5.1. N-linked glycosylation   

**Figure 4:**
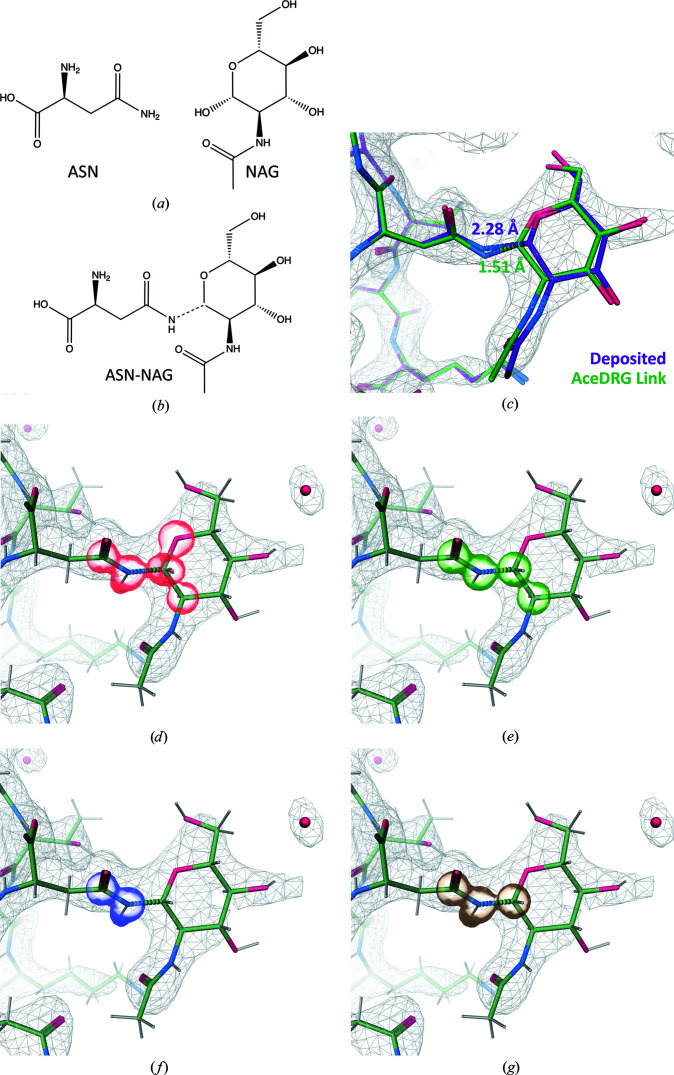
Description of the covalent linkage of *N*-acetylglucosamine (NAG) and asparagine (ASN) using *AceDRG*. (*a*) Chemical diagrams of the individual NAG and ASN components (from the CCP4-ML) and (*b*) the linked composite compound, in which the covalent linkage is depicted as a dotted line (created using *ChemDraw Professional* 17.1). (*c*) Comparison of a deposited 2.4 Å resolution model (PDB entry 3kwf; Mattei *et al.*, 2010 ▸; purple) and the model re-refined with *REFMAC*5 using an *AceDRG* link dictionary (green), focusing on NAG-A796 and ASN-A229, displayed using *Coot*. Interatomic distances and dotted lines corresponding to the linkage are shown for both models (note that the deposited model did not contain a corresponding link record). The 2*mF*_o_ − *DF*_c_ map corresponding to the re-refined model is shown as a grey mesh. Transparent surfaces surrounding atoms in the linked complex highlight the atoms involved in link-dictionary restraints, corresponding to (*d*) changes in bond/angle/chirality restraints (red surface), (*e*) torsion-angle restraints (green), (*f*) planar restraints that are removed (blue) and (*g*) planar restraints that are added (gold) due to the covalent linkage. H atoms were modelled in riding positions using *REFMAC*5.

There are 315 cases amongst 161 PDB entries in which the covalent linkage between *N*-acetylglucosamine (GlcNAc; NAG) and asparagine (ASN) was not modelled using a link record (noting that 32 927 such linkages are modelled amongst 6505 PDB entries). The N-linked glycosylation involves the removal of an O atom (O1) from NAG and the addition of a single bond between NAG[C1] and ASN[ND2]. Fig. 4 ▸ demonstrates the nature of this covalent linkage, and indicates which atoms are involved in the restraints that are updated as a consequence of the linkage (by *AceDRG*). Bond, angle, torsion and chirality restraints in the vicinity of the linkage are updated (Figs. 4 ▸ *d* and 4 ▸ *e*). Planarity restraints within both components are removed, and a new planar group involving both components is added (Figs. 4 ▸ *f* and 4 ▸ *g*). In the example model the covalent linkage is not modelled, and thus the interatomic distance between the linked atoms is unrealistically long (2.28 Å) due to repulsive forces during refinement. Re-refining the model using the *AceDRG* link dictionary results in an interatomic distance of 1.51 Å, which is closer to the target value of 1.431 Å (e.s.d. 0.011; Fig. 4 ▸ *c*). Whilst here we exemplify the manual modelling of a covalent linkage, note that *Coot* contains automated tools to facilitate the building of N-linked glycans (Emsley & Crispin, 2018 ▸), which are also applied automatically in *PDB-REDO* for the (re)building of N-linked glycans (van Beusekom *et al.*, 2019 ▸).

### 5.2. Covalent linkage of lysine and pyridoxal phosphate   

**Figure 5:**
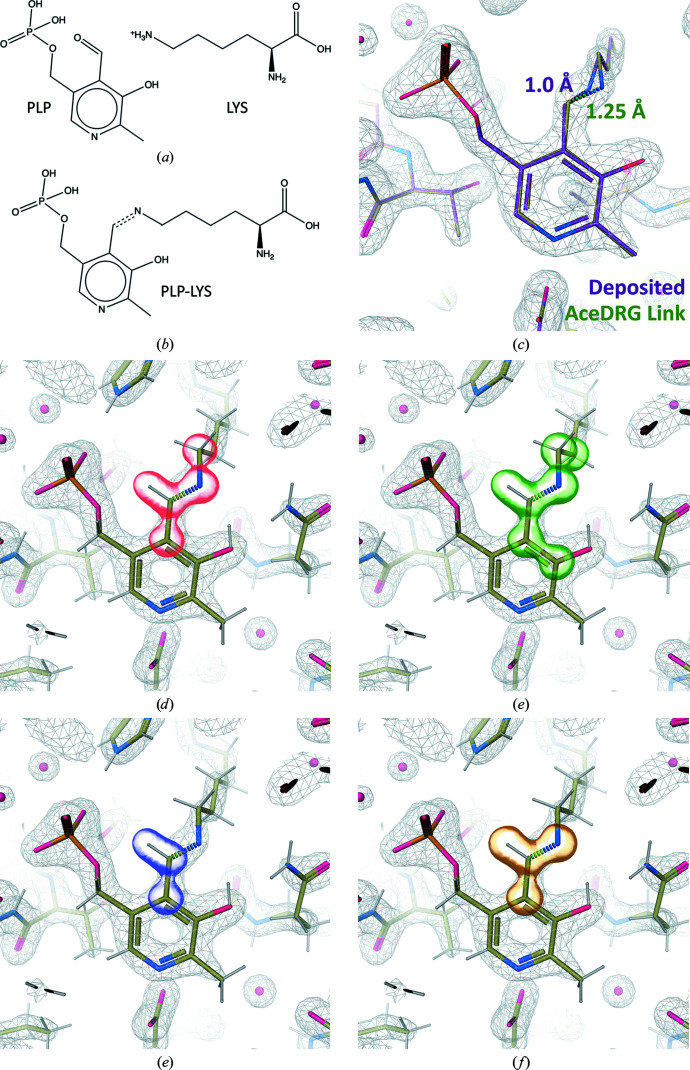
Description of the covalent linkage of lysine (LYS) and pyridoxal phosphate (PLP) using *AceDRG*. (*a*) Chemical diagrams of the individual LYS and PLP components (from the CCP4-ML) and (*b*) the linked composite compound, in which the covalent linkage is depicted as a dotted line (created using *ChemDraw Professional* 17.1). (*c*) Comparison of a deposited 1.8 Å resolution model (PDB entry 6ndn; J. F. Scortecci, J. Brandao-Neto, H. M. Pereira & O. H. Thiemann, unpublished work; purple) and the model re-refined with *REFMAC*5 using an *AceDRG* link dictionary (green), focusing on LYS-A226 and PLP-A501, displayed using *Coot*. Interatomic distances and dotted lines correspond to the covalent linkage. The 2*mF*_o_ − *DF*_c_ map corresponding to the re-refined model is shown as a grey mesh. Transparent surfaces surrounding atoms in the linked complex highlight the atoms involved in link-dictionary restraints, corresponding to (*d*) changes to bond/angle restraints (red surface), (*e*) torsion-angle restraints (green), (*f*) planar restraints that are removed (blue) and (*g*) planar restraints that are added (gold) due to the covalent linkage. Note that the O4A atom deleted from PLP (and thus not shown) was involved in the removed planar restraint. H atoms were modelled in riding positions using *REFMAC*5.

Fig. 5 ▸ demonstrates the covalent linkage of lysine (LYS) and pyridoxal phosphate (PLP). This reaction involves the removal of an O atom (O4A) from PLP and the addition of a double bond between LYS[NZ] and PLP[C4A] (Metzler, 2003 ▸). The link dictionary involves the addition of bond, angle and torsion restraints involving the linked atoms, as well as modifications of those in the immediate vicinity of the linkage (Figs. 5 ▸ *d* and 5 ▸ *e*). Planarity restraints are removed, and a new planar group involving both components is added (Figs. 5 ▸ *f* and 5 ▸ *g*). In the example model, the interatomic distance between the linked atoms is 1.0 Å (which is unrealistically short^6^). Re-refining the model without using a link record results in the interatomic distance increasing to 1.34 Å (which is unrealistically long), due to the atoms being subject to repulsive forces instead of being appropriately restrained during refinement. However, re-refinement using the *AceDRG* link dictionary results in an interatomic distance of 1.25 Å, which is close to the target value of 1.27 Å (e.s.d. 0.017; Fig. 5 ▸ *c*).

### 5.3. Modelling a methionine–tyrosine–tryptophan cross-link   

**Figure 6:**
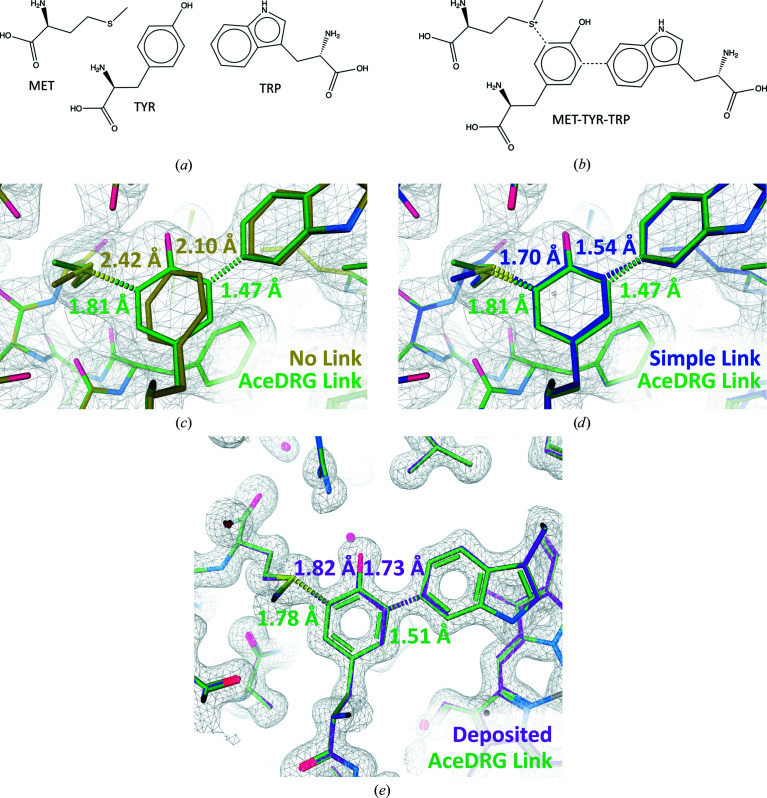
Description of the covalent linkages between methionine (MET), tyrosine (TYR) and tryptophan (TRP) in a MET–TYR–TRP cross-link; examples correspond to haem-dependent catalase–peroxidase enzymes. (*a*) Chemical diagrams of the individual MET, TYR and TRP components (from the CCP4-ML) and (*b*) the linked composite compound, in which the covalent linkages are depicted as dotted lines (created using *ChemDraw Professional *17.1). (*c*) and (*d*) show *Coot* depictions of a 2.4 Å resololution model (PDB entry 1sj2; Bertrand *et al.*, 2004 ▸) focused on MET-A255, TYR-A229 and TRP-A107 after re-refinement with *REFMAC*5. Models were re-refined without modelling the covalent linkage (*c*) (yellow), using an *AceDRG* link dictionary (*c*, *d*) (green) and using a link record but no dictionary (*d*) (blue). (*e*) *Coot* depiction of a 1.4 Å resolution model (PDB entry 5jhy; Gasselhuber *et al.*, 2016 ▸) focused on MET-A299, TYR-A273 and TRP-A140. The deposited model is shown (purple), as well as that after re-refinement with *REFMAC*5 using the *AceDRG* link dictionary (green). Interatomic distances between covalently linked atoms are shown and are coloured according to the corresponding model.

Fig. 6 ▸ exemplifies how the use of *AceDRG* link dictionaries facilitates the accurate modelling of a methionine–tyrosine–tryptophan (MET–TYR–TRP) cross-link. The first linkage is a single bond between MET[SD] and TYR[CE1], and the second is a single bond between TYR[CE2] and TRP[CH2].^7^ For brevity, we shall abbreviate these two linkages MET–TYR and TYR–TRP. Covalent linkage involves the addition of charge to the SD atom of MET, resulting in a sulfonium ion (Ghiladi *et al.*, 2005 ▸); the *AceDRG* link dictionary includes a description of this chemical modification.

Table 1 ▸ provides target restraint values along with the corresponding interatomic distances for two models of varying resolution refined without modelling the linkage, using a simple link record and using an *AceDRG* link dictionary. In the absence of a link dictionary, the target values for models with link records derive from the CCP4-ML (based on the covalent radii of the atoms). It is evident that there is a greater discrepancy between the default and *AceDRG* target values for MET–TYR than for TYR–TRP. This indicates that compared with the simple default covalent radii-based target values, the more detailed description of local stereochemistry adopted by *AceDRG* results in little difference to the linkage bond length for TYR–TRP but in a substantial difference in the case of MET–TYR (almost 0.2 Å). The latter exemplifies the utility of the more detailed and accurate description of stereochemistry provided by *AceDRG*.

In the 2.4 Å resolution model with PDB code 1sj2 (Figs. 6*c* and 6*d* ▸), failure to model the linkage results in the re-refined model exhibiting long interatomic distances for both linkages. This is due to repulsive forces during refinement, which also cause the aromatic ring in TYR to rotate out of position. The use of a link record, but without a link dictionary, results in interatomic distances that are closer to, but still noticeably greater than, the default target values that were used during refinement. This discrepancy warns of some internal inconsistency (between restraints and/or between model and experimental data) and thus potentially a suboptimal model. In contrast, using the *AceDRG* dictionary results in interatomic distances that are much closer to the respective refinement target values, indicating increased self-consistency.

Whilst the changes to coordinates resulting from the use of link dictionaries may be subtle, especially in cases where the data are of sufficiently high resolution to clearly indicate the position of each atom, the use of a more detailed dictionary nevertheless results in models that are more consistent with previous observations/prior knowledge (*i.e.* small-molecule models in the case of *AceDRG*). Fig. 6 ▸(*e*) shows the model with PDB entry 5jhy refined against higher resolution data (1.4 Å) using the same *AceDRG* link dictionaries.

As can be seen in Table 1 ▸, refinement without using a link record results in interatomic distances that are very similar to those in the deposited model (coloured purple in Fig. 6 ▸ *d*), indicating that the covalent linkage may not have been modelled in the original deposition. Re-refining the model with link records but without a link dictionary results in interatomic distances that are closer to the target values (the TYR-TRP linkage distance is affected more than MET-TYR), yet there is still a large discrepancy between the model and (default) dictionary values for both linkages. However, re-refinement using the *AceDRG* dictionary results in inter­atomic distances that are much more consistent with the *AceDRG* target values.

This highlights the importance of correctly modelling covalent linkages using comprehensive restraint dictionaries. Whilst the resultant effect on the coordinate parameters may be subtle, this treatment may be important for the subsequent interpretation and detailed analysis of interactions and strain. Here, we have focused purely on the interatomic distance corresponding to the covalent linkage itself, although in practice it may also be useful to analyse the behaviour of other geometric features in the linked components when determining an appropriate modelling strategy.

## 6. Discussion   

In this contribution, we have reviewed the mechanism for describing covalent linkages: the use of link-annotation records to specify the existence of link instances within a model, along with an appropriate restraint dictionary for each type of covalent linkage. We have described the process of link-dictionary generation using *AceDRG*, and have provided an overview of the various practical routes available for the modelling and application of covalent linkages within the *CCP*4 suite.

It is important to model covalent linkages using a sufficiently detailed link dictionary, which, in addition to containing inter-component stereochemical restraints, also reflects any changes to the individual components as a consequence of the reaction (*i.e.* modifications of the chemical composition of components and restraints describing intra-component stereochemistry). Such changes can have an effect on model geometry and thus subsequent interpretation, and so it is always advisable to use modelling assumptions (and restraints) that most accurately reflect the understanding of the chemistry within the crystal structure.

The examples provided in Section 5 demonstrate how the use of detailed link dictionaries facilitates the refinement of models in the presence of covalent linkages. Analysing the consistency of model configuration and restraint dictionaries can help to identify and thus avoid potential errors. However, such consistency analysis is alone insufficient, and should be complemented by more comprehensive validation of the model in the context of its structural environment, ensuring the favourability of interactions (Emsley, 2017 ▸).

When modelling covalent linkages, and in particular when generating link dictionaries, the user must specify the nature of the bonding. Such decisions (the removal/addition of atoms, the specification of bond orders and changes to formal charge) must be made manually, and thus care is needed when deciding linkage chemistry. Often, the MX data quality/resolution is insufficient to unambiguously determine appropriate chemistry, although inspecting discrepancies between model and density maps can provide diagnostic information by indicating potential errors. Referring to literature detailing the nature of a particular chemical reaction can aid this, noting that different environmental conditions can result in different chemistries (for example protonation states may vary with pH). In some cases complementary experiments and referring to higher resolution analogues may aid such decisions.

Whilst *AceDRG* can successfully be used to generate link dictionaries for the majority of covalent linkages, there are a number of scenarios that are currently unsupported; for example, when a covalent linkage (or the dictionary description) involves atoms from more than two components: there is presently no formal mechanism for dealing with this scenario in mmCIF restraint dictionaries. Notably, *AceDRG* cannot presently create dictionaries involving many metal-containing compounds (components must comprise only atoms with elemental types C, N, O, S, P, B, F, Cl, Br, I, H). Metals pose additional challenges, such as determining the coordination and analysing/describing environmental interactions. The ability to routinely and robustly create restraint dictionaries for metal-containing compounds is a future prospect. Also, care should be exercised in cases where a compound is involved in multiple covalent linkages.

We have discussed conventional approaches to modelling covalent linkages in *CCP*4 (Section 2). Whilst some other software adopt similar conventions, others may have different approaches; for example, implementation-specific treatment of ligand modifications and usage of the ‘link distance’ reported in link-annotation records. Such inconsistencies may cause undesirable behaviour when switching between different software suites during the structure-determination process. Another issue is the loss of linkage information upon deposition in the wwPDB: not only are the restraint dictionaries themselves omitted, but the (link) identifiers that reference the usage of a particular source of prior information are also discarded. This hampers subsequent model interpretation, analysis, model improvement and bioinformatics efforts. There is a need to have a unified convention for the treatment of component modifications and linkages and the use of link-annotation records in models, and to address communication and transfer of information about restraints used during the structure-determination process (metadata) to the wwPDB.

There is no one universal solution for modelling covalent linkages. Whilst some types are sufficiently common and well understood to be dealt with using automated solutions, for example pre-computed descriptions distributed in the CCP4-ML, the range of chemical configurations that might be encountered in MX means that manual intervention is often required. Consequently, users are encouraged to seek help from experts, who are keen to help and improve usability; user feedback facilitates the improvement of software tools, resources and interfaces. The responsibility for ensuring model quality is shared between the modeller/depositor (who should know the chemistry), software developers from different suites (who facilitate the process) and the wwPDB (who ensure the appropriate encapsulation of relevant information during deposition). Ensuring that all parties cooperate using a cohesive unified framework is a challenge. However, doing so is important in order to aid the quality and future interpretation of deposited models.

## Supplementary Material

Supplementary Figures. DOI: 10.1107/S2059798321001753/ir5021sup1.pdf

Link dictionaries generated by AceDRG, corresponding to the examples presented in Section 5. DOI: 10.1107/S2059798321001753/ir5021sup2.gz

## Figures and Tables

**Table 1:** Restraint-target values and the corresponding model interatomic distances for the MET–TYR and TYR–TRP covalent linkages in the models with PDB codes 1sj2 and 5jhy, as shown in Fig. 6 ▸ Target values correspond to the default value that is used by *REFMAC*5 in the absence of an explicit link dictionary and the value reported in the *AceDRG* link dictionary. Interatomic distances are shown for the deposited models, the models re-refined without a link record, re-refined with a link record but without a link dictionary and re-refined with an *AceDRG* link dictionary. Restraint-target estimated standard deviations (e.s.d.s) are shown in parentheses.

	MET–TYR	TYR–TRP
Target		
Default	1.610 Å (0.020)	1.460 Å (0.020)
*AceDRG*	1.795 Å (0.010)	1.486 Å (0.011)
PDB entry 1sj2 (2.4 Å)		
Deposited	1.73 Å	1.50 Å
Re-refined: no link	2.42 Å	2.10 Å
Re-refined: link record	1.70 Å	1.54 Å
Re-refined: *AceDRG* dictionary	1.81 Å	1.47 Å
PDB entry 5jhy (1.4 Å)		
Deposited	1.82 Å	1.74 Å
Re-refined: no link	1.82 Å	1.73 Å
Re-refined: link record	1.76 Å	1.62 Å
Re-refined: *AceDRG* dictionary	1.78 Å	1.51 Å

## Footnotes

1 Chain identifier in PDB files, and auth_asym_id in PDBx/mmCIF files. In order to avoid erroneous covalent linkages, links between atoms in different chains are not automatically created by default. For details of the monomer recognition and linkage algorithms in *REFMAC*5, see Vagin *et al.* (2004 ▸).

2 However, note that in practice there is no technical distinction between LINK and LINKR records. *REFMAC*5 will interpret link identifiers if present in the ‘link distance’ field, irrespective of whether they are presented in a LINK or LINKR record.

3 One relevant difference is that since PDB is a fixed-length format, the LINKR link identifier is restricted to eight characters, whereas in PDBx/mmCIF there is no such technical limitation.

4 This will undoubtedly have to change as the number of registered components rapidly approaches the three-character limit (47 988 possibilities): another reason necessitating migration to PDBx/mmCIF format for model-data storage.

5 Component and link identifiers cannot/should not be automatically assigned due to the requirement for consistency between atomic models and dictionaries, although modification record identifiers can be reassigned providing that the relevant link dictionaries are updated accordingly.

6 The deposited model includes a link record for this covalent linkage. However, it is not possible to infer the restraint target value that was used, as this information is lost upon deposition in the PDB.

7 Note that the CE1 and CE2 atoms in tyrosine are chemically equivalent, and thus may be interchanged. However, once link records have been defined the atoms should not be swapped. There should also be consistency between noncrystallographic symmetry (NCS)-related parts of the model.
