# Supplementary material for: Modelling covalent linkages in CCP4
Source: Acta Crystallogr D Struct Biol. 2021 May 19;77(Pt 6):712–26. doi: 10.1107/S2059798321001753 (PMC8171069; doi:10.1107/S2059798321001753)
Supplement: Supplementary file 1 [file d-77-00712-sup1.pdf]

STRUCTURAL
BIOLOGY

Volume 77 (2021)

Supporting information for article:

Modelling covalent linkages in *CCP*4

Robert A. Nicholls, Robbie P. Joosten, Fei Long, Marcin Wojdyr, Andrey Lebedev, Eugene Krissinel, Lucrezia Catapano, Marcus Fischer, Paul Emsley and Garib N. Murshudov

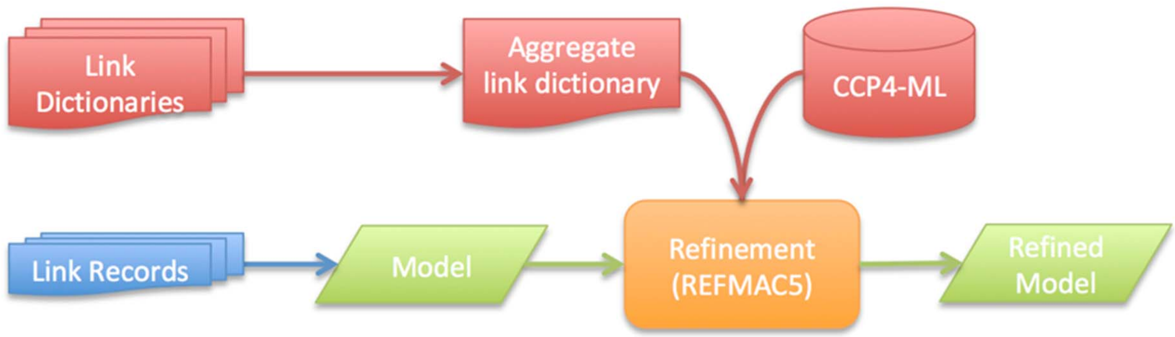

Figure S1 Linkage information dataflow in *CCP4*, focussing on the application of refinement with *REFMAC5*. The refinement process is depicted as an orange rectangle, data are represented as document symbols (arrays indicate potential presence of multiple instances), models as parallelograms, and the CCP4-ML databank as a cylinder. Arrows indicate directional flow, coloured according to the matching data type – red for link dictionaries, blue for link records, and green for models.

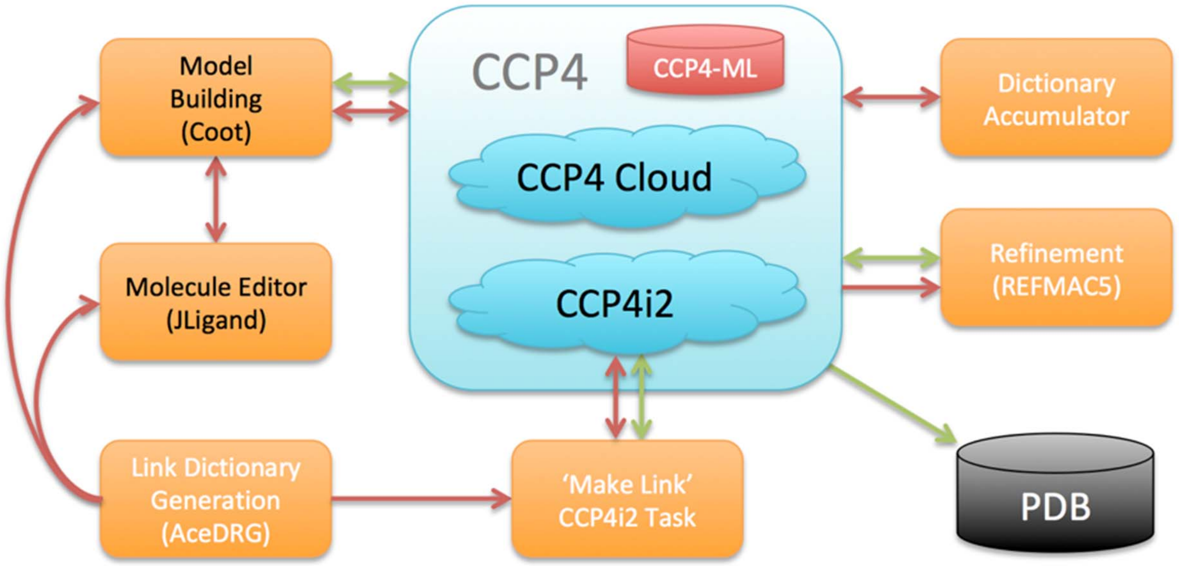

Figure S2 *CCP4* GUI-centric linkage process flow diagram. Processes are depicted as orange rectangles, databanks as cylinders, and GUIs as blue clouds. Arrows indicate directional flow, coloured according to the matching data type – red for link dictionaries, and green for models. Text labels are coloured black for graphical interactive processes, and white for (semi-)automated processes and data.
